# Malignancy rates for Bethesda III subcategories in thyroid fine needle aspiration biopsy (FNAB)

**DOI:** 10.6061/clinics/2018/e370

**Published:** 2018-05-19

**Authors:** Leticia Mosca, Luiz Fernando Ferraz da Silva, Paulo Campos Carneiro, Danielle Azevedo Chacon, Vergilius Jose Furtado de Araujo-Neto, Vergilius Jose Furtado de Araujo-Filho, Claudio Roberto Cernea

**Affiliations:** I Disciplina de Cirurgia de Cabeca e Pescoco, Departamento de Cirurgia, Faculdade de Medicina FMUSP, Universidade de Sao Paulo, Sao Paulo, SP, BR; II Departamento de Patologia, Faculdade de Medicina FMUSP, Universidade de Sao Paulo, Sao Paulo, SP, BR

**Keywords:** Thyroid, Ultrasound, Fine Needle Aspiration Biopsy, Bethesda

## Abstract

**OBJECTIVES:**

Most thyroid diseases are nodular and have been investigated using ultrasound-guided fine needle aspiration biopsy (FNAB), the reports of which are standardized by the Bethesda System. Bethesda category III represents a heterogeneous group in terms of lesion characteristics and the malignancy rates reported in the literature. The objective of the present study was to evaluate the differences in the malignancy rates among Bethesda III subcategories.

**METHODS:**

Data from 1,479 patients who had thyroid surgery were reviewed. In total, 1,093 patients (89.6% female, mean age 52.7 (13-89) years) were included, and 386 patients were excluded. FNAB results (based on Bethesda Class) and histopathological results (benign or malignant) for coincident areas were collected. Bethesda III patients were subcategorized according to cytopathological characteristics (FLUS: follicular lesion of undetermined significance, Bethesda IIIA; AUS: atypia of undetermined significance, Bethesda IIIB). Data were correlated to obtain the malignancy rates for each Bethesda category and the newly defined subcategory.

**RESULTS:**

FNAB results for these patients were as follows: Bethesda I: 3.1%; Bethesda II: 18.6%; Bethesda III: 35.0%; Bethesda IV: 22.1%; Bethesda V: 4.1%; and Bethesda VI: 17.1%. The malignancy rates for Bethesda Class IIIB were significantly higher than those for Bethesda Class IIIA (*p*<0.001) and Bethesda Class IV (*p*<0.001). Bethesda Class IIIA showed significantly lower malignancy rates than Bethesda Class III overall (*p*<0.001)

**CONCLUSIONS:**

Improvements of the Bethesda System should consider this subcategorization to better reflect different malignancy rates, which may have a significant impact on the decision-making process.

## INTRODUCTION

Most thyroid diseases occur in a nodular form and can reach a prevalence of up to 68% in adult women 1. Although most of these nodules are benign, the possibility of malignancy is a major concern. During 2017, more than 7,000 patients were diagnosed with thyroid cancer in Brazil, according to the National Institute of Cancer José Alencar Gomes da Silva (INCA) 2.

Ultrasound-guided fine needle aspiration biopsy (FNAB) is one of the most important tools to investigate thyroid nodules 3,4, and since 2009, FNAB reports have been standardized by the Bethesda System for Reporting Thyroid Cytopathology 5,6 (here referred only as Bethesda). Approximately 60% of all biopsied nodules are reported as benign, 10% present definitive criteria for malignancies, and 30% cannot be defined using only their cytological features 7. Bethesda class III is an important group that fits most of these undefined cases, and it is a heterogeneous category that includes nodules classified as a follicular lesion of undetermined significance (FLUS) or an atypia of undetermined significance (AUS). The expected malignancy rates for the Bethesda III category range from 5 to 15% according to relevant published studies 5. However, some recent papers have shown higher malignancy rates associated with AUSs 8-10, suggesting that the heterogeneity of lesions within this class may have relevant impacts on the diagnosis and management of patients.

The aim of this study was to evaluate the malignancy rates in two subcategories of Bethesda III at reference centers in Brazil.

## MATERIALS AND METHODS

The present study was approved by the institutional review board (CAPPESQ).

This retrospective study included 1,479 patients who underwent thyroidectomy at two university hospitals of the University of Sao Paulo Medical School (Hospital das Clínicas [HCFMUSP] and Instituto do Cancer do Estado de São Paulo [ICESP]), between September 2009 and April 2013. The study included a detailed review of clinical information from patients' hospital files, as well as from pathological samples (surgical specimens and FNAB slides) and pathological reports for all cases.

In order to better characterize this subset of patients in comparison to all patients undergoing FNAB in our centers (not only those that underwent thyroidectomy), we also reviewed all of the 4010 thyroid FNAB reports produced during the same period.

**Figure 1:**
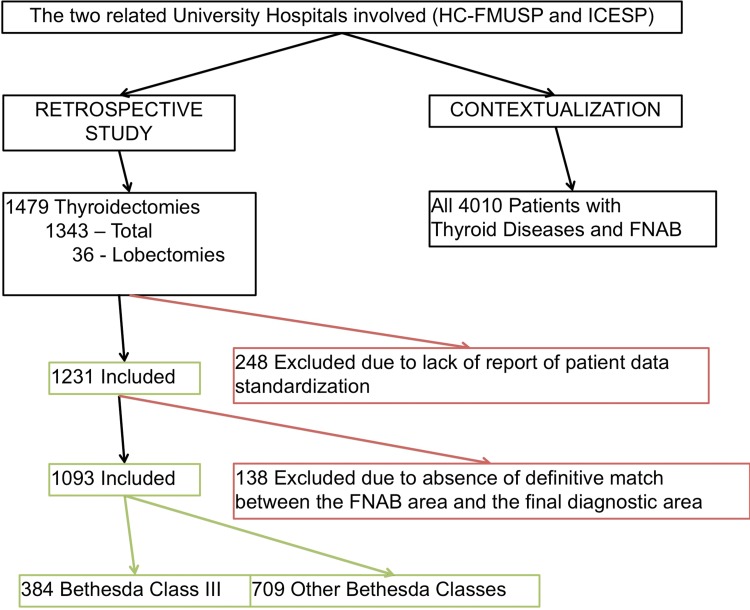
Schematic representation of the study design and patient inclusion/exclusion criteria.

For thyroidectomy patients, the inclusion criteria were as follows: (A) patients whose preoperative examinations, surgery and post-operative evaluations were all performed in the aforementioned institutions and (B) the availability of FNAB material for review. A flowchart depicting subject enrollment in this study according to the inclusion/exclusion criteria is presented in Figure 1.

From the 1,479 patients who underwent thyroidectomy, we excluded 386 patients (248 patients due to lack of reported patient data standardization and 138 due to the absence of a definitive match between the FNAB area and the post-operative tissue samples). Finally, there were 1,093 patients eligible for the study. Patients' files were reviewed by the medical team according to established standards of the Institutional Review Board (Ethics Committee), and reported FNAB data and final histological diagnoses were collected. When more than one biopsy was performed, the one higher with a Bethesda Class (higher malignancy risk) was considered for this study. We took extra care to specifically correlate the biopsied nodule to the final histological report (to compare the same reported areas), and the pathologists reviewing the histopathological report were blinded to the FNAB results and vice-versa.

Fine needle aspiration biopsy reports, as well as final histopathological reports from each patient, were reviewed and correlated to the corresponding tissue region and nodule. Patients diagnosed as Bethesda III were subdivided into two groups according to the cytological characteristics: Bethesda IIIA (FLUS) and Bethesda IIIB (AUS).

FLUSs were characterized by a small follicular pattern with poorly attached cells and a small amount of colloid. AUSs were characterized by the presence of cellular and nuclear atypias, regardless of the distribution pattern of the cells, which could be follicular, papillary or non-characteristic 5.

All of the patients underwent surgery for the classical indications of thyroidectomy (substernal goiter, compression, suspicious or confirmation of malignancy and hyperthyroidism of difficult clinical control).

Comparisons between malignancy rates in different categories were performed using chi-squared and Fisher's exact tests. Descriptive and statistical analyses were performed using SPSS 20 software (2011; IBM Corp, USA).

## RESULTS

**Figure 2:**
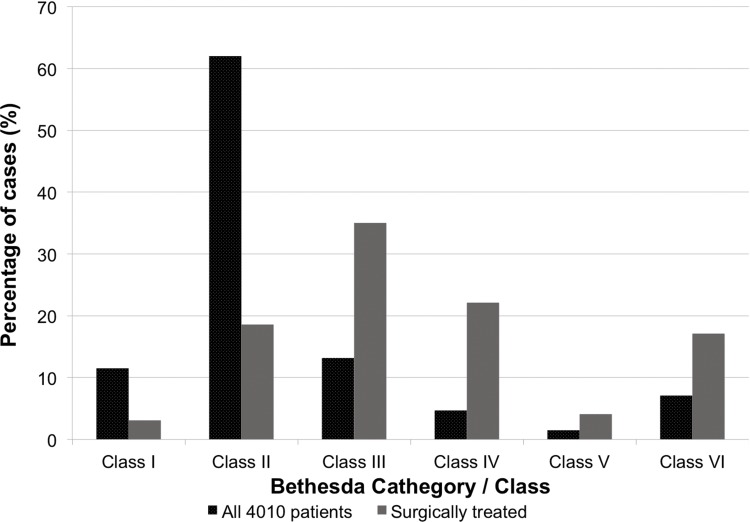
Distribution of Bethesda Classifications in FNAB reports in all patients and in the surgically treated patients.

There were 4,010 FNABs performed in our hospitals during the studied period. The frequency of each Bethesda Class was as follows: Bethesda I: 11.5% (463); Bethesda II: 62.0% (2481); Bethesda III: 13.2% (530); Bethesda IV: 4.7% (190); Bethesda V: 1.5% (62); and Bethesda VI: 7.1% (284) (Figure 2).

For the FNAB–thyroidectomy correlation and Bethesda III subcategorization, we included 1,093 patients who met the aforementioned inclusion and exclusion criteria. The studied population included 979 (89.6%) female patients. The mean age was 52.7 years (range: 13-89).

The FNAB results within these 1,093 cases included the following classifications: Bethesda I: 3.1% (34); Bethesda II: 18.6% (204); Bethesda III: 35.0% (384); Bethesda IV: 22.1% (242); Bethesda V: 4.1% (42); and Bethesda VI: 17.1% (187) (Figure 2).

The malignancy rates of the biopsied nodules as a function of Bethesda category are shown in Table 1.

The 384 subjects classified as Bethesda III were subcategorized according to histological results as Bethesda IIIA (FLUS) and IIIB (AUS). The malignancy rate for the 269 subjects classified as Bethesda IIIA (FLUS) was 5.6%, and the rate for the 115 subjects classified as Bethesda IIIB (AUS) was 27%. The absolute difference in the malignancy rates between the Bethesda IIIA and IIIB categories was 21.4% (95% CI 13-30) (Table 2). The absolute difference in the malignancy rates between the Bethesda IIIB subcategory (27%) and Bethesda IV subcategory (11.2%) was 16% (95% CI 7-25).

The distributions of the histopathological results in the patients undergoing operation according to FNAB subcategories are presented in Table 2. The malignancy rates for Bethesda IIIB were significantly higher than for Bethesda IIIA (*p*<0.001) and Bethesda IV (*p*<0.001) (Table 2). Bethesda IIIA cases had significantly lower malignancy rates than Bethesda III cases overall (*p*<0.001).

## DISCUSSION

Here, we present the association between FNAB and histopathological results in 1,093 patients who underwent thyroidectomy. We demonstrate that the malignancy rates within Bethesda Class III are heterogeneous and that these malignancy rates are significantly higher for Bethesda Class IIIB (AUS) than for Bethesda Class IIIA (FLUS).

In the Bethesda Classification System for Thyroid Lesions, Bethesda III is the most heterogeneous category because it merges follicular lesions without nuclear atypia (i.e., FLUSs) and lesions with nuclear atypia both in a follicular or a non-follicular pattern (i.e., AUSs). This combination obviously makes the lesions easy to classify as Class III, but it can also create a broad spectrum of diagnoses with different outcomes, which may make reaching a clinical decision difficult.

Therefore, we chose to divide this category into two subcategories: (1) Bethesda IIIA: FLUS and (2) Bethesda IIIB: AUS. The goal of this subcategorization was to demonstrate that the Bethesda III classification is very heterogeneous but that some of its characteristics and patterns can still be clearly defined on cytological smears and are related to a higher or lower risk of malignancy. These characteristics can thus be used to narrow the diagnostic spectrum and facilitate clearer and more cost-effective clinical decisions 8.

Since the implementation of the Bethesda System, there has been a decrease in non-diagnostic FNAB reports, which have become more accurate in providing descriptive interpretations of cytological findings 11. The FNA sensitivity reported in the literature varies from 65 to 99%, and its specificity varies from 72 to 100%. These values are strongly dependent on examiner experience and other technical details 12-14.

One of the most important features of the Bethesda System was the creation of Class III. This heterogeneous category includes cases that are neither clearly benign nor sufficiently characteristic to be classified into either of the two following categories: IV (follicular neoplasm or suspicious for follicular neoplasm) or V (suspicious for malignancy) 15. It involves some subjectivity, reflecting the difficulty in cytologically diagnosing follicular lesions, and this subjectivity is strongly associated with the interpretation of the pathologist 16,17.

The expected malignancy rate for Bethesda Class III ranges from 5 to 15% according to relevant studies 5. However, the actual malignancy rate in this subcategory, usually confirmed by histopathology, is very difficult to determine when not treated via surgery. In our study, the malignancy rate for Bethesda Class III was 12%, matching the previously expected malignancy rate.

Some studies have divided Bethesda III in two categories as follows: (a) cases presenting common characteristics of papillary carcinomas and (b) cases without features of papillary carcinoma. This study showed increased malignancy rates associated with the papillary features. 5. Another study from Renshaw subdivided Bethesda III in 4 groups, and found a difference in the malignancy risks between two of them; the group with papillary carcinoma characteristics had a 38% higher risk of malignancy 18.

Recently, other authors have divided Bethesda III nodules into those with low cellularity with a microfollicular pattern and those showing nuclear atypia, and some studies have suggested that nuclear atypia is an independent risk factor. Kelman et al. found that up to 60% of thyroid nodules with nuclear atypia were related to malignant disease, while only 7% of those without atypia had the same diagnosis 19,20.

Other authors have evaluated the subclassification of Bethesda III in the following two categories: architectural atypia and nuclear atypia. Gan et al. 9 showed that patients with nuclear atypia had a malignancy rate of 35% compared to a rate of 10% for other Bethesda III cases. Similarly, Shrestha et al. 10 showed a 36.8% malignancy rate for patients with nuclear atypia compared to rate of 10.8% in those without nuclear atypia. Another recent study from Brazil, analyzing results from second FNABs in patients with a prior Bethesda III diagnosis showed a malignancy rate of 41.5% in patients with nuclear atypia, while those with architectural atypia had a malignancy rate of 15.5% 21.

In our study, we found a malignancy rate of 27% for Bethesda IIIB cases, which is lower than the observed malignancy rates for cases with nuclear atypia in previous studies. We also found a malignancy rate of 5.6% for Bethesda IIIA cases, which is also lower than the rates observed for published cases of architectural atypia. One possible explanation for these lower rates, which is also a unique aspect of this study, is the fact that we took special care to correlate FNAB results with the tissue sample of the same biopsied nodule, without considering the diagnosis in the whole post-operative specimen.

Interestingly our malignancy rates for Bethesda IIIB were higher than the upper expected limit for Bethesda III in the literature (15%). Additionally, this rate was significantly higher than the malignancy rates expected for Bethesda IV in the literature 8 and the rate the observed for our own Bethesda IV cases (11.2%).

Several authors have demonstrated the heterogeneity of Bethesda Class III. The subcategorization criteria and nomenclature vary, but in the end, they all lead to the same key message: Malignancy rates can be associated with certain patterns. In this study, we preferred to use the nomenclature IIIA and IIIB for two reasons: (1) we believe the single letters better fit the numbers used for Bethesda (instead of including the acronyms) and (2) it leaves room for future developments to include in the same subcategory (A or B) all the relevant features (architectural organization, nuclear atypia, cytoplasm alteration, etc.) that proved to be related to increased malignancy rates, thus helping to standardize diagnoses and reports.

In conclusion, the higher malignancy rates systematically observed in cases with nuclear atypia and its related features demonstrate that improvements in the Bethesda System for thyroid FNAB reports should consider the subcategorization of Class III in order to better reflect these differences and positively impact the decision-making process for further diagnostic tests and/or treatment selection.

## AUTHOR CONTRIBUTIONS

Mosca L was responsible for the data collection, case review and data analysis. Silva LF was responsible for the data analysis and manuscript writing. Carneiro PC was responsible for the study design, case review and manuscript review. Chacon DA was responsible for the data collection and case review. Araujo-Neto VJ was responsible for the manuscript writing. Araujo-Filho VJ was responsible for the study design and manuscript review. Cernea CR was responsible for the manuscript review.

## Figures and Tables

**Table 1:** Malignancy rates of biopsied nodules as a function of Bethesda category.

FNAB	Benign	Malignant	Cases
Bethesda I	94.1%	5.9%	34
Bethesda II	98.5%	1.5%	204
Bethesda III	88%	12%	384
Bethesda IV	88.8%	11.2%	242
Bethesda V	26.2%	73.8%	42
Bethesda VI	1.1%	98.9%	187

**Table 2:** Malignancy rates of related biopsied nodule as a function of Bethesda III subcategory.

	Benign	Malignant	Cases
Bethesda III (overall)	88%	12%	384
Bethesda IIIA	94.4%^a^	5.6%^a^	269
Bethesda IIIB	73%^b^	27%^b^	115
Bethesda IV	88.8%	11.2%	242

a *p*<0.001 compared with III (overall).

b *p*<0.001 compared with IIIA and IV.
